# Safety, pharmacokinetics, and pharmacodynamics of efzimfotase alfa, a second-generation enzyme replacement therapy: phase 1, dose-escalation study in adults with hypophosphatasia

**DOI:** 10.1093/jbmr/zjae128

**Published:** 2024-08-13

**Authors:** Kathryn M Dahir, Amy Shannon, Derek Dunn, Walter Voegtli, Qunming Dong, Jawad Hasan, Rajendra Pradhan, Ryan Pelto, Wei-Jian Pan

**Affiliations:** Program for Metabolic Bone Disorders, Vanderbilt University Medical Center, Nashville, TN 37232-8148, United States; Development, Regulatory and Safety, Alexion, AstraZeneca Rare Disease, Boston, MA 02210, United States; Development, Regulatory and Safety, Alexion, AstraZeneca Rare Disease, Boston, MA 02210, United States; Development, Regulatory and Safety, Alexion, AstraZeneca Rare Disease, Boston, MA 02210, United States; Development, Regulatory and Safety, Alexion, AstraZeneca Rare Disease, Boston, MA 02210, United States; Development, Regulatory and Safety, Alexion, AstraZeneca Rare Disease, Boston, MA 02210, United States; Development, Regulatory and Safety, Alexion, AstraZeneca Rare Disease, Boston, MA 02210, United States; Bioanalytical and Biomarker Development, Alexion, AstraZeneca Rare Disease, New Haven, CT 06510, United States; Development, Regulatory and Safety, Alexion, AstraZeneca Rare Disease, Boston, MA 02210, United States

**Keywords:** alkaline phosphatase, clinical trial, efzimfotase alfa, enzyme replacement therapy, diseases and disorders of/related to bone, other, therapeutics, other, hypophosphatasia, tissue-nonspecific alkaline phosphatase

## Abstract

Hypophosphatasia (HPP) is a rare, inherited metabolic disease caused by deficient activity of tissue-nonspecific alkaline phosphatase (TNSALP). Efzimfotase alfa (ALXN1850) is a second-generation TNSALP enzyme replacement therapy in development for HPP. This first-in-human open-label, dose-escalating phase 1 trial evaluated efzimfotase alfa safety, tolerability, pharmacokinetics, pharmacodynamics, and immunogenicity. Fifteen adults (5/cohort) with HPP received efzimfotase alfa in doses of 15 mg (cohort 1), 45 mg (cohort 2), or 90 mg (cohort 3) as one intravenous (i.v.) dose followed by 3 weekly subcutaneous (s.c.) doses. The primary objective was to assess safety and tolerability. Secondary objectives included pharmacokinetics, pharmacodynamics of ALP substrates known to be biomarkers of disease (inorganic pyrophosphate [PPi] and pyridoxal 5′-phosphate [PLP]) and immunogenicity. Treatment-emergent adverse events (TEAEs) occurred in 12 (80%) participants. Eight (53%) participants had injection site reactions (ISRs), observed after 10 of 41 (24%) s.c. injections. Most ISR TEAEs were mild and resolved within 1–2 d. Peak and total exposures of efzimfotase alfa increased in a greater-than-dose proportional manner over the range of 15–90 mg after i.v. and s.c. dosing. The arithmetic mean elimination half-life was approximately 6 d; absolute bioavailability was 28.6%–36.8% over the s.c. dose range of 15–90 mg. Dose-dependent reductions in plasma concentrations of PPi and PLP relative to baseline reached nadir in the first week after i.v. dosing and were sustained for 3–4 wk after the last s.c. dose. Four (27%) participants tested positive for antidrug antibodies (ADAs), 3 of whom were ADA positive before the first dose of efzimfotase alfa. ADAs had no apparent effect on efzimfotase alfa pharmacokinetics/pharmacodynamics. No participants had neutralizing antibodies. Efzimfotase alfa demonstrated acceptable safety, tolerability, and pharmacokinetic profiles and was associated with sustained reductions in biomarkers of disease in adults with HPP, supporting further evaluation in adult and pediatric patients.

**Registration:** ClinicalTrials.gov NCT04980248 (https://clinicaltrials.gov/study/NCT04980248).

## Introduction

Hypophosphatasia (HPP) is a rare, inherited metabolic disease caused by deficient activity of tissue-nonspecific alkaline phosphatase (TNSALP), a cell-surface phosphohydrolase expressed mainly in the bone, kidney, and liver.^1^ Diminished TNSALP activity promotes extracellular accumulation of the enzyme’s substrates, including inorganic pyrophosphate (PPi), a potent inhibitor of bone mineralization, and pyridoxal 5′-phosphate (PLP), the active coenzyme form of vitamin B_6_.^2^^-^^4^ A deficiency of TNSALP enzyme activity and the accumulation of its substrates can lead to impaired bone mineralization (osteomalacia or rickets), fractures and pseudofractures that heal poorly, impaired mobility, abnormal gait, musculoskeletal pain, chondrocalcinosis, muscle weakness, pain, fatigue, premature loss of deciduous teeth and other dental abnormalities, abnormal calcium and phosphate metabolism (eg, hypercalcemia), and renal manifestations (hypercalciuria and nephrocalcinosis).^3^^,^^5^^-^^8^ Many clinical manifestations are similar between pediatric and adult patients, although some differences exist, particularly in infants with HPP. Affected infants often present with respiratory complications, craniosynostosis, vitamin B_6_-responsive seizures, failure to thrive, widespread impaired bone mineralization, rickets, and bone deformities.^6^^,^^9^^,^^10^

Asfotase alfa (STRENSIQ®; Alexion, AstraZeneca Rare Disease), the first recombinant TNSALP enzyme replacement therapy (ERT) for treating HPP,^11^^,^^12^ was developed to address the underlying cause of HPP by supplementing the deficient enzyme, thus correcting mineralization defects of the skeleton and improving bone health and other systemic manifestations in patients with HPP. In 2015, asfotase alfa received approval in the USA and the European Union for the treatment of pediatric-onset HPP^11^^,^^12^ and in Japan for the treatment of HPP regardless of the age of onset.^13^ The recommended dosage is 6 mg/kg per week administered via the subcutaneous (s.c.) route as either 2 mg/kg/dose 3 times per week or 1 mg/kg/dose 6 times per week based on studies in predominantly pediatric populations.^11^^,^^12^ A subsequent phase 2a, randomized, open-label study confirmed the dosage of 6 mg/kg per week for the treatment of adults with HPP.^14^

**Figure 1:**
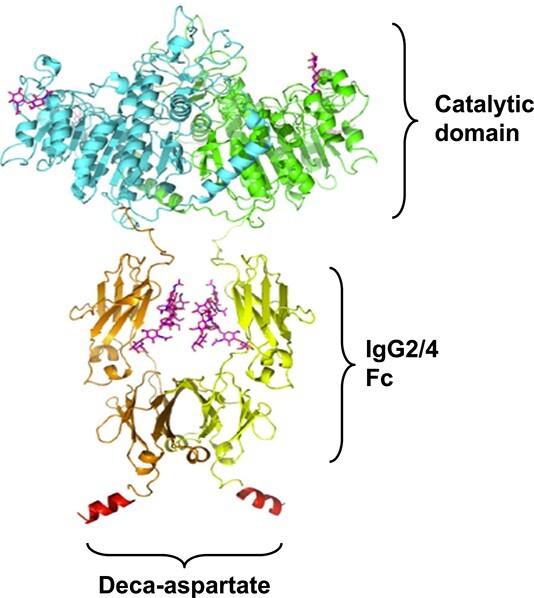
Structure of efzimfotase alfa. Efzimfotase alfa is a soluble Fc fusion protein with a molecular weight of about 160 kDa and comprises 2 polypeptide chains covalently linked by 2 disulfide bonds. Each polypeptide chain contains 724 amino acids and comprises 3 segments. The first segment, the N-terminal region L1-A491, contains the soluble part of the human tissue-nonspecific ALP enzyme and contains the catalytic function. Within the enzymatic region of each polypeptide chain, a single point mutation (E108M) was introduced to increase enzymatic activity and 2 N-linked glycosylation sites removed via asparagine-to-glutamine mutations (N213Q and N286Q) for further improving compound PK. The second portion contains amino acids V492-K714 and comprises the Fc part of the human IgG2/4 containing hinge, CH2, and CH3 domains. The third part, the C-terminal region of the polypeptide chain, contains 10 aspartic acids in a peptide sequence derived from bone-binding proteins. Abbreviations: ALP, alkaline phosphatase; Fc, fragment crystallizable; IgG2/4, immunoglobulin gamma-2/4.

Efzimfotase alfa (ALXN1850) is a second-generation TNSALP ERT in clinical development for the treatment of HPP (Patent US 2021/0169994 A1).^15^ Structurally related to asfotase alfa, efzimfotase alfa has undergone several structural modifications (Figure 1). As such, efzimfotase alfa is a soluble glycoprotein that consists of 2 polypeptide chains of 724 amino acids comprising 3 segments: the catalytic domain of human TNSALP; a human IgG2/4 fragment crystallizable (Fc) domain to facilitate purification and extend half-life (t_½_); and a deca-aspartate peptide, which enhances affinity to the bone. These structural modifications have the potential to enhance enzymatic activity in substrate hydrolysis and improve the pharmacokinetic (PK) profile. In vitro, efzimfotase alfa hydrolyzes PPi approximately twice as fast as asfotase alfa. Hydrolysis activity for PLP is also higher for efzimfotase alfa than for asfotase alfa.^15^ The structural modifications in efzimfotase alfa and improvements in its posttranslational modifications support lower doses and reduce injection volume and dosing frequency compared with asfotase alfa, all of which may translate to fewer injection site reactions (ISRs) and improved patient treatment experience.

The objectives of this first-in-human, open-label, dose-escalating study were to assess the safety, tolerability, PK, pharmacodynamics (PD), and immunogenicity of efzimfotase alfa when given by intravenous (i.v.) and s.c. routes to adults with HPP. The study was designed to identify an efficient, tolerable, and pharmacologically effective dose of efzimfotase alfa based on its PK characteristics in participants with HPP.

## Materials and methods

### Participants

Eligible participants were adults (≥18 yr old) with a clinical diagnosis of HPP who were not expected to require ERT after study completion based on their baseline signs and symptoms and the investigator’s clinical assessment. Participants were required to have a confirmed *ALPL* gene variant that was pathogenic (P) or likely pathogenic (LP) or a variant of uncertain significance (VUS) and ALP plasma activity below the laboratory age- and sex-adjusted normal range (age ≥ 19 yr, males and females: 40–150 U/L). Genetic testing was conducted by Clinical Laboratory Improvement Amendments-approved laboratories (Invitae, Baylor Genetics, or PreventionGenetics). Participants were excluded if they had primary or secondary hyperparathyroidism or hypoparathyroidism; a fracture within 12 wk of screening; a current or relevant history of unstable physical or psychiatric illness; significant allergies; asfotase alfa use within 6 mo; or a positive test result for asfotase alfa antidrug antibodies (ADAs) after prior treatment with asfotase alfa (participants without prior exposure to asfotase alfa were not tested for asfotase alfa ADAs at screening), or neutralizing antibodies (NAbs) at screening. Female participants who were pregnant, planning to become pregnant, or breastfeeding were excluded.

The study was conducted in accordance with the ethical principles of Good Clinical Practice and the International Council for Harmonisation Guidelines. The protocol and informed consent form were approved by the institutional review board of the investigative site. All participants provided informed consent before study initiation.

### Study design

**Figure 2:**
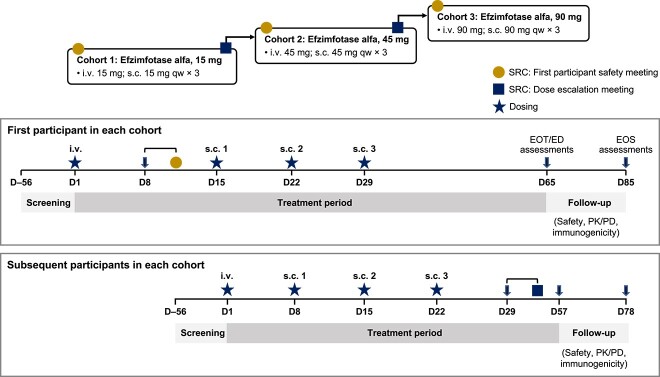
Study design. Abbreviations: D, day; ED, early discontinuation; EOS, end-of-study; EOT, end-of-treatment; i.v., intravenous; PD, pharmacodynamics; PK, pharmacokinetics; qw, weekly; s.c., subcutaneous; SRC, safety review committee.

This open-label, dose-escalation phase I trial (NCT04980248) was conducted at 2 sites in the USA from August 16, 2021 to August 24, 2022. Eligible participants were enrolled into 3 cohorts (cohort 1: efzimfotase alfa 15 mg; cohort 2: efzimfotase alfa 45 mg; and cohort 3: efzimfotase alfa 90 mg) (Figure 2). First-in-human dose selection was based on PK data collected from a study in WT mice, dose range–finding studies in rats and monkeys, 4-wk good laboratory practice toxicology studies in rats and monkeys, and efficacy data from mouse models of HPP. A no-adverse-effect-level–based approach was used to determine the starting dose (15 mg i.v. and s.c.) with a subsequent typical 3-fold dose escalation step to 45 mg i.v. and s.c. Given the projected substantial reduction in plasma PPi, the highest efzimfotase alfa dose was escalated at a 2-fold step to 90 mg i.v. and s.c. Efzimfotase alfa dosed at 15, 45, or 90 mg without weight-based adjustments was projected to normalize bone mineralization in 66%, 85%, and 91% of the HPP patient population, respectively, based on dose–response modeling of the mouse efficacy study results. Population PK analysis with dose-exposure response supports a flat-dose approach for efzimfotase alfa treatment.

After the first participant in cohort 1 (15 mg) had received a single i.v. dose of efzimfotase alfa, a safety review committee (SRC) convened to assess safety and tolerability and to endorse the initiation of s.c. administration in the first participant and initiation of i.v. and s.c. administration in additional participants in that cohort. All s.c. doses were administered once weekly for 3 wk in the abdomen. Cohorts 2 (45 mg) and 3 (90 mg) began dosing after the SRC had reviewed all relevant safety, tolerability, and PK data from at least 3 participants in cohort 1 or cohorts 1 and 2, as well as endorsed dose escalation. All participants were offered enrollment in the Global HPP Registry after completion of the study for safety follow-up. A total of 15 eligible participants were to be enrolled to allow for a minimum of 12 (4/cohort) to complete the study, with the sample size determined empirically. Amendments to the trial protocol were minor and made to address recommendations from regulatory agencies.

### Outcome measures

The primary objective was to assess the safety and tolerability of efzimfotase alfa given i.v. as a single dose and s.c. in 3 weekly doses based on the incidence of treatment-emergent adverse events (TEAEs) and serious TEAEs.

The study had 4 secondary objectives. The first was to assess the PK of efzimfotase alfa based on efzimfotase alfa activity over time. The second was to assess the absolute bioavailability (F) of efzimfotase alfa based on area under the plasma concentration vs time curve during the dosing interval (AUC_tau_) after i.v. and s.c. administration. The third was to measure the PD effects of efzimfotase alfa on plasma concentrations of the TNSALP substrates PPi and PLP and the PLP/pyridoxal (PL) ratio over time. The fourth was to evaluate the immunogenicity potential of efzimfotase alfa based on the observed incidences of ADAs and NAbs.

Exploratory outcomes included analyses of the effects of efzimfotase alfa on bone homeostasis and related markers, including changes in ionized calcium, phosphorus, magnesium, PTH, serum C-terminal telopeptide of type 1 collagen (sCTX-1), P1NP, osteocalcin, and pyridoxic acid over time.

### Safety assessments

TEAEs were monitored continuously throughout the study and were coded using the Medical Dictionary for Regulatory Activities version 25.0. TEAEs were deemed related or unrelated to study drug by investigator judgment. ISRs were assessed by the investigator and were defined as TEAEs localized to the site of i.v. or s.c. administration, occurring at any time during the study. Clinical site personnel also recorded ISRs on an injection site evaluation form; some ISRs reported on this form were not captured as TEAEs and are described below in the Results section. Injection-associated reactions (IARs) were defined as treatment-related systemic TEAEs (eg, fever, chills, flushing, heart and blood pressure alterations, dyspnea, nausea, vomiting, diarrhea, generalized skin rashes) that occurred during i.v. or s.c. dosing or within 24 h after the start of drug administration.

### Pharmacokinetic assessments

Blood samples for the measurement of plasma efzimfotase alfa activity were collected before each i.v. and s.c. dose; at 2, 4, 8, 12, 24, 48, 72, 96, and 168 h after each i.v. and s.c. dose; and on days 51, 56, and 85 for the first participant in each cohort or on days 43, 57, and 78 for subsequent participants in each cohort. Plasma levels of efzimfotase alfa were determined by Charles River Laboratories using an ALP enzyme activity assay^16^ with para-Nitrophenylphosphate as the substrate that was previously validated for the measurement of asfotase alfa activity.^16^ The limits of assay quantification ranged from 0.141 to 2.20 μg/mL.

Plasma efzimfotase alfa PK parameters evaluated included maximum concentration (C_max_), time to C_max_ (t_max_) post-dosing, and AUC from time 0 to the last quantifiable concentration (AUC_t_) or AUC_tau_ after the single i.v. dose and each of the 3 s.c. doses; AUC from time 0 to time infinity (AUC_∞_), total body clearance (CL), and volume of distribution (Vd) after the i.v. dose; trough (predose) concentration (C_trough_) before each of the 3 s.c. doses; apparent clearance (CL/F) and apparent volume of distribution (Vd/F) after the third s.c. dose; terminal elimination t_½_ after the i.v. dose and the third s.c. dose; the s.c. accumulation ratio (R; third dose/first dose); and the absolute bioavailability after the third s.c. dose.

### Pharmacodynamic assessments

Blood samples for the measurement of plasma PPi, PLP, and PL concentrations were collected before each i.v. and s.c. dose; at 24, 48, 72, 96, and 168 h after each i.v. and s.c. dose; and on days 51, 56, and 85 for the first participant in each cohort or on days 43, 57, and 78 for subsequent participants in each cohort. Samples were treated with 100 mM levamisole (one part levamisole to 9 parts matrix [final concentration 10 mM levamisole]) during collection to inhibit ALP activity related to efzimfotase alfa administration. Efficacy of levamisole was validated in the PPi assay using human plasma with or without the addition of levamisole prior to whole blood collection. Blood samples collected for PPi analysis were centrifuged at 2500*g* for 15 min, and the resulting plasma was filtered through a 0.2-μm centrifugal filter (Sartorius #VS0671) prior to freezing for removal of platelets. Plasma samples were analyzed for PPi concentrations (lower limit of quantification [LLOQ]: 0.75 μM; upper limit of quantification [ULOQ]: 12.00 μM) by Charles River Laboratories using previously described methods.^14^ Plasma levels of PLP (LLOQ: 5.00 ng/mL; ULOQ: 500 ng/mL) and PL (LLOQ: 2.00 ng/mL; ULOQ: 200 ng/mL) were analyzed by PPD Laboratory Services.

### Assessment of anti-efzimfotase alfa antibodies

Antibodies against efzimfotase alfa in serum samples were detected and measured using an electrochemiluminescence immunoassay carried out by PPD Laboratory Services.

### Statistical analysis

All participants who received at least a single dose of efzimfotase alfa were included in the safety analysis population. Safety data were summarized using descriptive statistics. All treated participants were included in the PK analysis population; PK data collected after missed s.c. doses were excluded. PD data were reported for all participants after i.v. dosing up to day 5, and only data for subsequent participants (ie, those who were not the first participant treated in each cohort) are reported after the first s.c. dose; PD data collected after missed doses were excluded. Noncompartmental PK parameters were determined from individual plasma efzimfotase alfa concentration data using Phoenix WinNonlin 8 or higher (Certara). Concentrations that were below the assay LLOQ and were associated with timepoints before the first quantifiable concentration in a profile were replaced with 0 for the noncompartmental analyses. Absolute bioavailability of efzimfotase alfa after s.c. dosing was assessed by comparing AUC_tau_ values of the third s.c. dose vs AUC_∞_ of the i.v. dose for the same dose cohorts. AUC parameters were calculated using log-linear trapezoidal methods. Log-transformed AUC_tau_ and AUC_∞_ estimates were analyzed using an analysis of variance model with group (i.v. vs. s.c.) as a factor. The parameter estimates results were exponentiated, and the ratio of geometric means and 90% CIs was calculated. The dose proportionality of plasma efzimfotase alfa exposures was assessed across the dose range of 15 to 90 mg via a power model using log of C_max_ and AUC as response variables and log of dose as a fixed effect. Attainment of steady state was evaluated by assessing the relationship between C_trough_ and time using a linear mixed regression model with participants as a random effect. This approach involved calculating the 95% CI around the slope estimate and identifying the first instance at which 0 was included in the 95% CI, indicating that steady state was reached. The arithmetic mean (SD) values for plasma PPi and PLP concentrations and PLP/PL ratios after efzimfotase alfa administration are presented by time. Individual plasma PPi, PLP, and PL concentrations that were below the assay LLOQs (0.75 μM, 5 ng/mL, and 2 ng/mL, respectively) were calculated as 50% of the LLOQs for the computation of summary statistics. Statistical comparisons were not performed on PD or exploratory outcomes.

## Results

### Participants

Of the 23 participants with HPP who provided informed consent, 15 met eligibility criteria and subsequently received at least one dose of efzimfotase alfa; all 15 completed the study (Figure S1). Three participants missed a total of 4 doses because of asymptomatic COVID-19 infection, including 1 participant in cohort 2 who missed 2 s.c. doses and 2 participants in cohort 3 who missed 1 s.c. dose each. Participants were predominantly female (73%), White (100%), and had a median age of 47.0 yr (range: 26–64 yr) (Table 1). All 15 participants had an *ALPL* variant detected by genetic sequencing, most commonly c.1250A > G (*n* = 4), c.648 + 1G > A (*n* = 3), and c.1231A > G (*n* = 2) (Table 2); 13 participants had 14 P variants, 1 had an LP variant, and 1 had a VUS.

**Table 1:** Baseline participant characteristics.

**Characteristic**	**Cohort 1: 15 mg** **(*n* = 5)**	**Cohort 2: 45 mg** **(*n* = 5)**	**Cohort 3: 90 mg** **(*n* = 5)**	**Total** **(*N* = 15)**
**Sex, *n* (%)**				
**Female**	3 (60)	3 (60)	5 (100)	11 (73)
**Male**	2 (40)	2 (40)	0	4 (27)
**Race, White, *n* (%)**	5 (100)	5 (100)	5 (100)	15 (100)
**Ethnicity, not Hispanic or Latino, *n* (%)**	5 (100)	5 (100)	5 (100)	15 (100)
**Age (yr)**				
**Mean (SD)**	44.2 (12.4)	37.8 (12.5)	54.6 (9.1)	45.5 (12.8)
**Median (min, max)**	42.0 (30, 57)	31.0 (26, 55)	57.0 (40, 64)	47.0 (26, 64)
**Height (cm)**				
**Mean (SD)**	170.3 (8.2)	171.5 (13.6)	166.1 (7.3)	169.3 (9.6)
**Median (min, max)**	169.0 (162.8, 183.0)	171.0 (155.0, 192.0)	165.0 (159.0, 178.0)	166.9 (155.0, 192.0)
**Weight (kg)**				
**Mean (SD)**	87.5 (11.7)	93.5 (32.2)	71.2 (16.7)	84.1 (22.6)
**Median (min, max)**	87.8 (74.7, 100.4)	84.4 (64.5, 137.4)	66.8 (56.8, 97.8)	76.7 (56.8, 137.4)
**BMI (kg/m^2^)**				
**Mean (SD)**	30.2 (3.2)	31.3 (7.7)	25.8 (6.1)	29.1 (6.1)
**Median (min, max)**	29.2 (26.2, 33.7)	35.1 (22.3, 38.3)	23.3 (21.1, 35.9)	28.6 (21.1, 38.3)
**ALP at screening (IU/L)**				
**Mean (SD)**	26.8 (10.4)	23.6 (2.1)	24.6 (5.9)	25.0 (6.6)
**Median (min, max)**	31.0 (9, 35)	24.0 (21, 26)	25.0 (16, 30)	25.0 (9, 35)
**Baseline PLP (ng/mL)**				
**Mean (SD)**	160.7 (249.9)	50.4 (21.0)	40.8 (14.8)	83.9 (145.6)
**Median (min, max)**	62.1 (32.2, 607.0)	43.4 (31.0, 84.9)	41.7 (21.8, 57.3)	43.4 (21.8, 60.7)
**Baseline PPi (μM)**				
**Mean (SD)**	3.0 (0.82)	3.0 (0.52)	3.0^a^ (0.55)	3.0^b^ (0.60)
**Median (min, max)**	2.6 (2.4, 4.4)	2.8 (2.5, 3.9)	3.0^a^ (2.3, 3.6)	2.8^b^ (2.3, 4.4)

a *n* = 4, one sample not analyzed due to hemolysis.

b *n* = 14, one sample not analyzed due to hemolysis.

Abbreviations: ALP, alkaline phosphatase; BMI, body mass index; IU, international units; max, maximum; min, minimum; PLP, pyridoxal 5′-phosphate; PPi, inorganic pyrophosphate; SD, standard deviation.

**Table 2:** *ALPL* variants.

**Cohort** **Participant**	**Variant (pathogenicity)**	
	**Allele 1**	**Allele 2**
**Cohort 1**		
**Pt #1**	c.876_881delAGGGGA (P)	None
**Pt #2**	c.876_881del (P)	None
**Pt #3**	c.648 + 1G > A (P)	None
**Pt #4**	c.526G > A (P)	c.891C > A (P)
**Pt #5**	c.648 + 1G > A (P)	None
**Cohort 2**		
**Pt #6**	c.1250A > G (P)	None
**Pt #7**	c.1250A > G (P)	None
**Pt #8**	c.648 + 1G > A (P)	None
**Pt #9**	c.1231A > G (P)	None
**Pt #10**	c.1231A > G (P)	None
**Cohort 3**		
**Pt #11**	c.130C > T (LP)	None
**Pt #12**	c.1250A > G (P)	None
**Pt #13**	Deletion of exon 2 (P)	None
**Pt #14**	c.1250A > G (P)	None
**Pt #15**	c.1156G > T (VUS)	None

Abbreviations: LP, likely pathogenic; P, pathogenic; VUS, variant of uncertain significance.

### Safety

#### Treatment-emergent adverse events

A total of 46 TEAEs occurred in 12 (80%) participants, including 3 of 5 (60%) in cohort 1 (15 mg), all 5 (100%) in cohort 2 (45 mg), and 4 of 5 (80%) in cohort 3 (90 mg) (Table 3). Across cohorts, the TEAEs reported in at least 2 participants were COVID-19 (*n* = 3; 20%), headache (*n* = 3; 20%), fatigue (*n* = 2; 13%), injection site bruising (*n* = 2; 13%), injection site erythema (*n* = 2; 13%), hyperphosphatemia (*n* = 2; 13%), anemia (*n* = 2; 13%), and hematuria (*n* = 2; 13%). The majority of TEAEs were grade 1 or 2 in severity. Grade 3 TEAEs occurred in one participant in cohort 2 (hypertension) and one in cohort 3 (serious TEAE of atrial fibrillation that occurred 28 d after the last dose of efzimfotase alfa). The latter event was not observed on repeat electrocardiogram and was deemed unrelated to the study drug by the investigator. No events consistent with hypersensitivity reactions or anaphylaxis occurred. There were no discontinuations or dose modifications because of TEAEs, and no deaths occurred during the study.

**Table 3:** Summary of treatment-emergent adverse events.

	**Cohort 1: 15 mg**			**Cohort 2: 45 mg**			**Cohort 3: 90 mg**			**Total**		
***n* (%) of participants** **[no. of events]**	**Overall** **(*n* = 5)**	**i.v.** **(*n* = 5)**	**s.c.** **(*n* = 5)**	**Overall** **(*n* = 5)**	**i.v.** **(*n* = 5)**	**s.c.** **(*n* = 5)**	**Overall** **(*n* = 5)**	**i.v.** **(*n* = 5)**	**s.c.** **(*n* = 5)**	**Overall** **(*N* = 15)**	**i.v.** **(*n* = 15)**	**s.c.** **(*n* = 15)**
**Any TEAE**	3 (60) [12]	2 (40) [2]	3 (60) [10]	5 (100) [26]	5 (100) [7]	5 (100) [19]	4 (80) [8]	2 (40) [4]	4 (80) [4]	12 (80) [46]	9 (60) [13]	12 (80) [33]
**Related**	3 (60) [8]	2 (40) [2]	3 (60) [6]	5 (100) [19]	5 (100) [6]	4 (80) [13]	2 (40) [2]	1 (20) [1]	1 (20) [1]	10 (67) [29]	8 (53) [9]	8 (53) [20]
**Serious TEAE**	0	0	0	0	0	0	1 (20) [1]	0	1 (20) [1]	1 (7) [1]	0	1 (7) [1]
**ISR TEAEs**	2 (40) [2]	0	2 (40) [2]	2 (40) [6]	0	2 (40) [6]	1 (20) [1]	0	1 (20) [1]	5 (33) [9]	0	5 (33) [9]
**Injection site bruising**	1 (20) [1]	0	1 (20) [1]	1 (20) [1]	0	1 (20) [1]	0	0	0	2 (13) [2]	0	2 (13) [2]
**Injection site erythema**	0	0	0	1 (20) [3]	0	1 (20) [3]	1 (20) [1]	0	1 (20) [1]	2 (13) [4]	0	2 (13) [4]
**Injection site ecchymosis**	0	0	0	1 (20) [1]	0	1 (20) [1]	0	0	0	1 (7) [1]	0	1 (7) [1]
**Injection site pruritus**	0	0	0	1 (20) [1]	0	1 (20) [1]	0	0	0	1 (7) [1]	0	1 (7) [1]
**ISR NOS**	1 (20) [1]	0	1 (20) [1]	0	0	0	0	0	0	1 (7) [1]	0	1 (7) [1]
**Potential IARs**	2 (40) [3]	1 (20) [1]	2 (40) [2]	2 (40) [4]	1 (20) [2]	2 (40) [2]	1 (20) [1]	1 (20) [1]	0	5 (33) [8]	3 (20) [4]	4 (27) [4]

Abbreviations: IAR, injection-associated reaction; ISR, injection site reaction; NOS, not otherwise specified; TEAE, treatment-emergent adverse event.

#### Treatment-related adverse events

Treatment-related TEAEs, including ISRs, occurred in 3 (60%) participants in cohort 1 (15 mg), 5 (100%) in cohort 2 (45 mg), and 2 (40%) in cohort 3 (90 mg). Treatment-related TEAEs reported by more than 1 participant overall included headache (*n* = 3) and fatigue, injection site bruising, injection site erythema, and hematuria (*n* = 2 each). Treatment-related TEAEs reported by 1 participant each included injection site hemorrhage, injection site pruritus, ISR, vessel puncture site bruise, arthralgia, muscle spasms, spinal pain, urinary tract infection, vulvovaginal mycotic infection, hyperphosphatemia, hypoglycemia, anemia, abdominal pain, gamma-glutamyltransferase increase, and hyperphosphaturia.

One participant experienced a TEAE consistent with a potential IAR within 24 h of i.v. administration (mild headache considered related to treatment by the investigator).

#### Injection site reactions

Treatment-related TEAEs were mainly related to ISRs. Across all 3 cohorts, 8 (53%) participants had ISRs after 10 of the 41 (24%) s.c. injections. Five of the 15 (33%) participants had ISRs, all grade 1 or 2 in severity (Table 3), and 3 additional participants had ISRs reported by clinical personnel on the injection site evaluation form that were not reported as TEAEs. Among the 8 participants with any ISR, 6 had a single ISR event (mild erythema [*n* = 4], mild soreness/swelling/redness [*n* = 1], and mild bruising [*n* = 1]) and 2 had more than 1 ISR (erythema and bruising after the first s.c. injection [*n* = 1] and erythema after the first s.c. injection, erythema and pruritus after the second s.c. injection, and erythema and ecchymosis after the third s.c. injection [*n* = 1]). The timing of ISRs with s.c. administration followed no clear pattern: 3 participants had ISRs only after the first injection, 2 only after the second injection, and 2 only after the third injection; one participant had reactions after each s.c. injection. Most ISRs resolved within 1 to 2 d. ISRs that took more than 2 d to resolve included bruising (resolved after 7 d [*n* = 1]); soreness, swelling, and redness (resolved after 4 d [*n* = 1]); and erythema (resolved after 13 d [*n* = 1]).

### Pharmacokinetics of efzimfotase alfa

**Figure 3:**
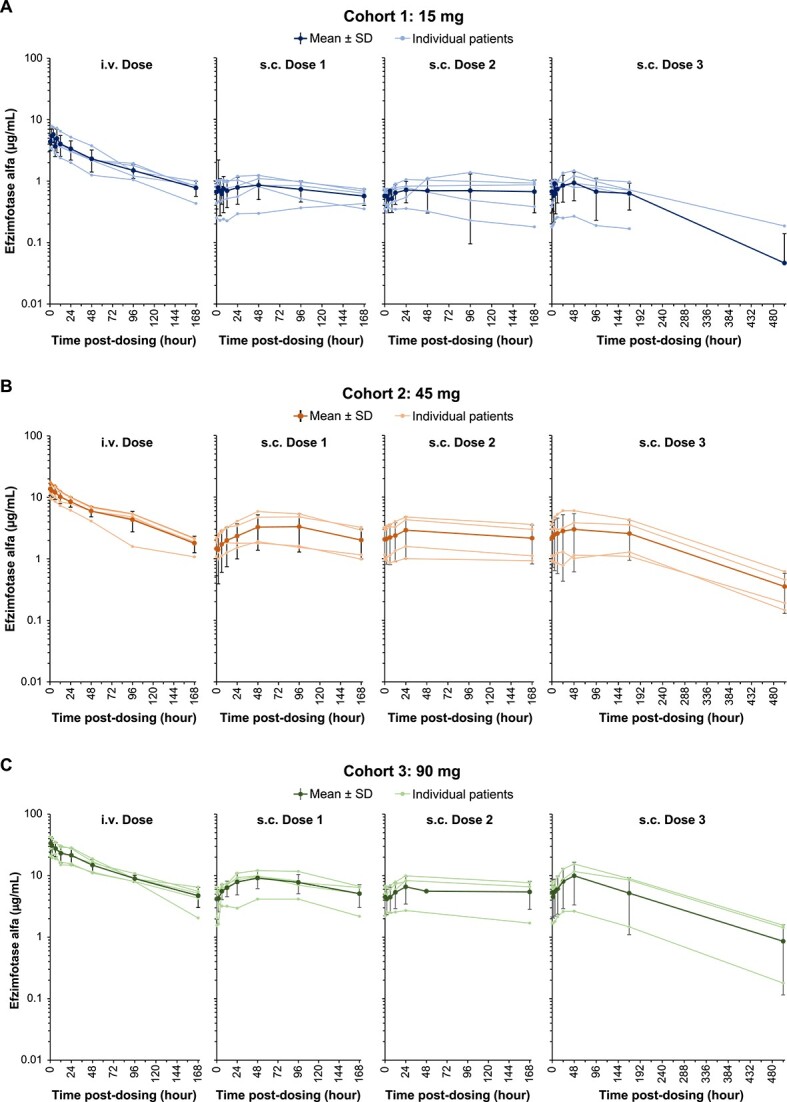
Plasma efzimfotase alfa concentrations in individual participants and mean ± SD in all participants after a single i.v. dose and each of the 3 s.c. doses in (A) cohort 1 (15 mg), (B) cohort 2 (45 mg), and (C) cohort 3 (90 mg). Data are plotted on a semilogarithmic scale. Data obtained after missed doses are not shown. Abbreviations: i.v., intravenous; s.c., subcutaneous; SD, standard deviation.

After the single i.v. and each of the 3 subsequent s.c. doses administered at 1-wk intervals, the mean plasma concentration vs time profiles in each dose cohort were stable and followed similar parallel trends without a clearly discernible distribution phase (Figure 3). Median t_max_ after i.v. administration was reached at 2.00, 0.60, and 0.72 h for cohorts 1, 2, and 3, respectively, relative to the 0.5-h i.v. infusion duration (Table 4). The median t_max_ for each cohort was approximately 48 h, 24 h, and 36 to 48 h after the first, second, and third s.c. doses, respectively. The concentration-time profiles showed increased plasma efzimfotase alfa exposures with increasing dose (Figure 3). Peak (C_max_) and total exposures (AUC) of efzimfotase alfa increased with dose in a greater than dose-proportional manner over the dose range of 15–90 mg. The 90% CIs of the slope for C_max_ and AUC were not contained within the criteria for dose proportionality (0.875–1.12) (Table S1). Over the dose range evaluated, the arithmetic means of the observed terminal half-lives throughout the limited study duration, t_½_ ranged from 66.7 to 78.0 h after a single i.v. dose and 135 to 136 h (approximately 6 d) after the third weekly s.c. dose (Table 4). Geometric mean CL after i.v. dosing was 0.04 L/h in each cohort. The geometric mean CL/F after the third s.c. dose ranged from 0.08 to 0.13 L/h across cohorts. Geometric mean Vd ranged from 3.7 to 4.3 L with i.v. dosing, and Vd/F ranged from 9.7 to 21.3 L with the third s.c. dose. The absolute bioavailability after the third s.c. dose was 28.6% (90% CI, 10.5%–78.2%), 36.7% (18.0%–74.8%), and 36.8% (9.1%–149%) for cohort 1 (15 mg), cohort 2 (45 mg), and cohort 3 (90 mg), respectively (Table S2). Linear regression of plasma C_trough_ on days 8, 15, and 22 demonstrated attainment of steady state on day 8 in each cohort, as 0 was included in the 95% CI of the regression slopes (Table S3).

**Table 4:** Plasma efzimfotase alfa pharmacokinetic parameters.

**Dose**	**Parameter** ^a^	**Cohort 1: 15 mg**	**Cohort 2: 45 mg**	**Cohort 3: 90 mg**
**i.v. dose**		**(*n* = 5)**	**(*n* = 5)**	**(*n* = 5)**
	C_max_ (μg/mL)	5.31 (29.2)	13.5 (23.3)	32.4 (31.1)
	t_max_ (h)^b^	2.00 (0.63, 2.02)	0.60 (0.53, 1.95)	0.72 (0.67, 0.83)
	AUC_168_ (h•μg/mL)	324 (34.1)	838 (23.7)	2010 (28.7)
	AUC_∞_ (h•μg/mL)	407 (38.3)	1010 (26.0)	2450 (33.6)
	t_½_ (h)^c^	78.0 (12.4)	66.7 (6.96)	73.1 (23.5)
	CL (L/h)	0.0369 (38.3)	0.0448 (26.0)	0.0367 (33.6)
	Vd (L)	3.98 (48.0)	4.29 (15.6)	3.73 (41.4)
**s.c. dose 1**		**(*n* = 5)**	**(*n* = 4)**	**(*n* = 5)**
	C_max_ (μg/mL)	0.871 (43.8)	3.16 (65.1)	8.60 (43.5)
	C_trough_ (μg/mL)	0.589 (62.5)	1.75 (33.7)	3.59 (72.7)
	t_max_ (h)^b^	48.03 (4.00, 168.00)	47.98 (0, 71.17)	48.33 (47.70, 72.32)
	AUC_tau_ (h•μg/mL)	115 (42.2)	416 (63.7)	1110 (49.6)
**s.c. dose 2**		**(*n* = 5)**	**(*n* = 4)**	**(*n* = 4)**
	C_max_ (μg/mL)	0.815 (52.3)	2.38 (88.8)	6.53 (64.9)
	C_trough_ (μg/mL)	0.543 (32.9)	1.80 (67.8)	4.67 (55.9)
	t_max_ (h)^b^	24.02 (4.17, 167.02)	24.00 (23.88, 24.22)	24.07 (23.82, 24.15)
	AUC_tau_ (h•μg/mL)	111 (58.5)	350 (83.3)	875 (84.2) [*n* = 3]
**s.c. dose 3**		**(*n* = 5)**	**(*n* = 4)**	**(*n* = 3)**
	C_max_ (μg/mL)	0.844 (75.2)	2.41 (97.4)	7.82 (121)
	C_trough_ (μg/mL)	0.558 (86.1)	1.82 (77.3)	4.41 (100.0)
	t_max_ (h)^b^	47.98 (0, 48.08)	35.95 (23.92, 48.33)	48.25 (48.17, 48.40)
	AUC_tau_ (h•μg/mL)	115 (73.9)	367 (95.6)	1060 (119)
	t_½_ (h)^c^	NA	136 (7.68) [*n* = 2]	135 (3.97) [*n* = 2]
	CL/F (L/h)	0.131 (73.9)	0.123 (95.6)	0.0848 (119)
	Vd/F (L)	NA	21.3 (134) [*n* = 2]	9.65 (13.1) [*n* = 2]
	R	0.998 (78.0)	0.882 (28.8)	1.00 (39.9)
	F (%)	28.0 (134) [*n* = 4]	36.7 (66.5)	44.1 (67.2)

a Values are geometric mean (geometric CV%) unless otherwise noted.

b Values for t_max_ are median (range).

c Values for t_½_ are arithmetic mean (SD).

Terminal elimination phase–dependent parameters (AUC_∞_, CL/F, and Vd/F) were not estimated where terminal elimination phase was not clearly defined (ie, AUC_extrap_ > 20%). Data available following missed s.c. doses were excluded.

AUC_∞_, area under the plasma concentration vs time curve from time 0 to infinity; AUC_168_, area under the plasma concentration vs time curve from time 0 to 168 h; AUC_extrap_, extrapolated area under the plasma concentration vs time curve; AUC_tau_, area under the plasma concentration vs time curve from time 0 to dosing interval; CL, total body clearance; CL/F, apparent total clearance; C_max_, maximum observed plasma concentration; C_trough_, plasma trough (predose) concentration observed at the start of the dosing interval; CV, coefficient of variation; F, absolute bioavailability; R, s.c. accumulation ratio (third s.c. dose/first s.c. dose); NA, not available; t_½_, terminal elimination half-life; t_max_, time to maximum observed plasma concentration; Vd, volume of distribution; Vd/F, apparent volume of distribution.

### Pharmacodynamics of efzimfotase alfa

#### Inorganic pyrophosphate

**Figure 4:**
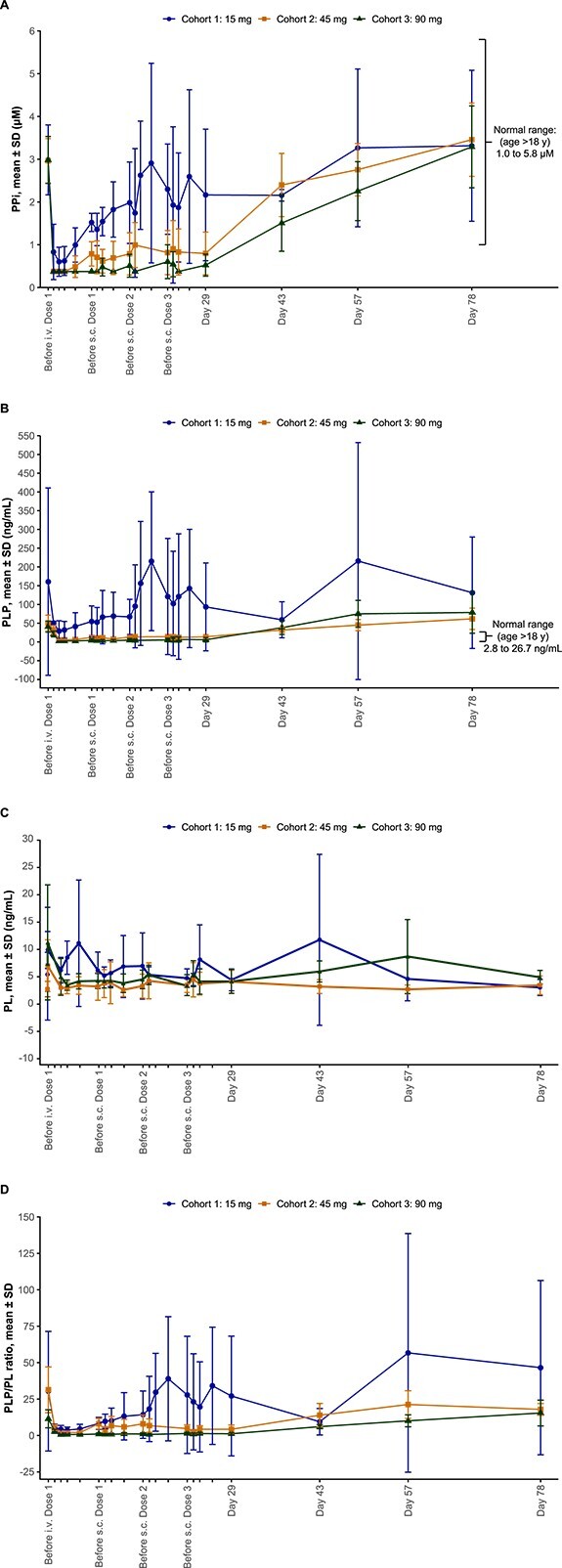
Mean (A) PPi concentrations, (B) PLP concentrations, (C) PL concentrations, and (D) PLP/PL ratio in plasma over time after a single i.v. dose and 3 s.c. doses of efzimfotase alfa. Baseline is represented as day 1, before administration of the i.v. dose. Data include all participants after the i.v. dose up to day 5. Data from the first participant in each cohort were excluded from the analysis after the first s.c. dose. Data obtained after missed doses were also excluded. Mean values calculated by setting individual plasma PPi concentrations that were below the assay LLOQ to 50% of the LLOQ (0.75 μM) are shown. Abbreviations: i.v., intravenous; LLOQ, lower limit of quantification; PL, pyridoxal; PLP, pyridoxal 5′-phosphate; PPi, inorganic pyrophosphate; s.c., subcutaneous; SD, standard deviation.

Baseline (day 1, before i.v. infusion) mean (SD) plasma PPi levels were similar between cohorts (cohort 1: 2.98 [0.82] μM; cohort 2: 2.96 [0.52] μM; and cohort 3: 2.98 [0.55] μM) and were within the normal ranges for adults >18 yr old (1.00–5.82 μM), ranging from 2.30 to 4.39 μM in all participants. The mean plasma PPi concentrations were ≥60% lower at day 5 (4 d after i.v. dosing) than at baseline and remained ≥45% lower before the first s.c. dose on day 8 (7 d after i.v. dosing) (Figure 4A). Within the first 7 d after i.v. dosing, plasma PPi concentrations were below the assay LLOQ (0.75 μM) in 36% (for 1–3 d), 80% (for 2–7 d), and 100% (for 7 d) of the samples after the 15 mg, 45 mg, and 90 mg doses, respectively. When values below the assay LLOQ were included in mean calculations, the plasma PPi profiles showed mostly zero (or below assay LLOQ) over the first 7 d after the 45 mg and 90 mg i.v. doses. Mean values calculated by setting individual plasma PPi concentrations that were below the assay LLOQ to 50% of the LLOQ (0.75 μM) are shown in Figure 4A. The reductions in plasma PPi concentrations were maintained after each s.c. dose, and the magnitude and duration of these reductions appeared to be dose dependent, with mean values maintained within the normal range in the 15-mg and 45-mg dose cohorts and falling below the lower limit of normal as well as assay LLOQ at most timepoints after s.c. dosing in the 90-mg dose group (Figure 4A). Mean plasma PPi levels remained below baseline values for 3 to 5 wk after the last s.c. dose (days 43 and 57) and gradually returned to baseline values by day 78 (study completion). All patients had detectable PPi levels at study completion.

#### Pyridoxal 5′-phosphate

Mean (SD) baseline plasma PLP levels were above the upper limit of normal for adults ≥18 yr old (2.8 − 26.7 ng/mL) in cohort 1 (160.7 [249.9] ng/mL), cohort 2 (50.4 [21.0] ng/mL), and cohort 3 (40.8 [14.8] ng/mL). Within the first 7 d after i.v. administration, plasma PLP concentrations were below the assay LLOQ (5 ng/mL) in 0%, 12% (for 3 d), and 72% (for 3–7 d) of the samples after the 15 mg, 45 mg, and 90 mg doses, respectively. Mean values calculated by setting individual plasma PLP concentrations that were below the assay LLOQ to 50% of the LLOQ (5 ng/mL) are shown in Figure 4B. Mean plasma PLP concentrations decreased by ≥69% from baseline, and these reductions were dose-dependent, falling within the normal range 1 d after i.v. dosing (days 2–5 in Figure 4B). Plasma PLP concentrations were maintained within the normal range after 45 mg and 90 mg s.c. doses, gradually returning to baseline values at days 43 and 57, 3 to 5 wk after the last s.c. dose (Figure 4B). All patients had detectable PLP levels at study completion.

#### Pyridoxal

Mean (SD) baseline plasma PL levels were similar among patients in cohort 1 (5.4 [4.1] ng/mL), cohort 2 (2.8 [1.4] ng/mL), and cohort 3 (7.4 [10.3] ng/mL). Mean values calculated by setting individual PL concentrations below the assay LLOQ to 50% of the LLOQ (2 ng/mL) are shown in Figure 4C. PL concentrations transiently increased by 50% in cohort 1, by 144% in cohort 2, and by 107% in cohort 3 immediately after the first i.v. dose (Figure 4C) and returned to baseline by day 2 (Figure 4C). Subsequent s.c. dosing on days 8, 15, and 22 did not consistently affect mean plasma PL levels, with increases or decreases in PL detected 1 d after dosing across cohorts (Figure 4C). PL concentration was detectable for each patient at study completion.

#### Pyridoxal 5′-phosphate/pyridoxal

Changes in the plasma PLP/PL ratio over time (Figure 4D) were similar to those observed for PLP (Figure 4B). Mean values determined by setting individual plasma PLP and PL concentrations that were below the assay LLOQs to 50% of the LLOQ (5 ng/mL and 2 ng/mL for plasma PLP and PL, respectively) are shown in Figure 4D. The mean PLP/PL ratio was ≥73% lower at day 5 (4 d after i.v. dosing) than at baseline, decreasing in a dose-dependent manner. Decreases in the PLP/PL ratio were maintained after 45 mg and 90 mg s.c. doses, with the ratio being sustained below baseline values for up to 5 wk after the last s.c. dose (Figure 4D).

### Anti-efzimfotase alfa antibodies

Four (27%) participants tested positive for anti-efzimfotase alfa ADAs in serum during the study, 3 of whom were ADA positive at baseline (without prior exposure to asfotase alfa). One participant (7%) from cohort 1 (15 mg) had a treatment-emergent ADA response, first testing positive for anti-efzimfotase alfa ADAs on day 51 of the study. Of note, this participant had a pre-existing autoimmune disease (lupus). All ADA-positive participants had low titers (range: 10–80). No participants were positive for NAbs at any time during the study. There was no apparent effect of ADA on efzimfotase alfa PK/PD.

### Exploratory biomarkers

Overall, no clinically meaningful trends in changes over time were noted for ionized calcium, phosphorus, magnesium, PTH, sCTX-1, P1NP, osteocalcin, or pyridoxic acid relative to baseline values (Figure S2). Hyperphosphatemia was reported in 2 participants in cohort 1, and elevated blood phosphorus was observed in one participant in cohort 3. All of these TEAEs were considered resolved. One participant with no evidence of high phosphate levels prior to study entry developed transient hyperphosphatemia that was considered a treatment-related TEAE by the investigator.

## Discussion

This first-in-human clinical study demonstrated that efzimfotase alfa given i.v. as a single dose and s.c. once weekly for 3 wk across the dose range of 15 to 90 mg had an acceptable safety profile and was well tolerated in adults with HPP. TEAEs were mostly limited to skin reactions at the injection site that resolved quickly (within 1–2 d). The safety and tolerability profile of efzimfotase alfa was consistent with that reported for asfotase alfa in previous clinical studies, in which TEAEs were also limited mainly to ISRs.^14^^,^^17^

Because of structural similarities between efzimfotase alfa and asfotase alfa, the PK profiles of these agents would be anticipated to be in line with each other on a dose and bodyweight-normalized basis.^16^ However, accurate comparisons between efzimfotase alfa and asfotase alfa PK studies are not possible because of differences in the attributes across studies between the 2 development programs (eg, differences in bioanalytical methods).^16^ The systemic peak and total exposures to efzimfotase alfa and asfotase alfa both increased with increasing dose^16^; however, systemic exposures to efzimfotase alfa increased in a greater than dose-proportional manner over the dose range evaluated (15–90 mg). Although acknowledging the differences in data attributes between the 2 development programs, it is noteworthy that dose- and bodyweight-normalized plasma total exposure (AUC) was much higher after dosing with efzimfotase alfa compared with exposure reported in a similarly designed first-in-human study of asfotase alfa (NCT00739505), with dose-adjusted values being approximately 21-fold higher after i.v. administration and 17-fold higher after s.c. administration.^18^ The higher dose-normalized systemic exposure suggests that efzimfotase alfa clinical efficacy could be equally maintained at lower dosing levels or with less frequent dosing than asfotase alfa.

The plasma PK profile of efzimfotase alfa observed in the present study accumulated evidence from studies with asfotase alfa, and the close similarities between efzimfotase alfa and asfotase alfa collectively justify the undertaking of future clinical trials of efzimfotase alfa in larger cohorts of pediatric and adult patients with HPP. Three clinical trials (NCT06079359, NCT06079281, and NCT06079372) assessing the efficacy and safety of efzimfotase alfa in pediatric (>2 yr of age), adolescent, and adult patients with HPP are underway. Further investigation of the clinical benefits of efzimfotase alfa in neonates, infants, and toddlers (0–2 yr of age) with HPP may be initiated to assess efficacy and safety in this high-risk population.

Efzimfotase alfa administration resulted in dose-dependent reductions in plasma levels of TNSALP substrates, including PPi and PLP. These results align with those of a randomized open-label, multicenter phase 1 clinical study in which 6 mo of s.c. administration of 2.1 or 3.5 mg/kg per week asfotase alfa significantly reduced plasma PPi and PLP concentrations compared with no treatment in adults with HPP.^17^ Similar effects of asfotase alfa on these disease biomarkers were observed in infants and young children with life-threatening or debilitating perinatal or infantile HPP.^19^ Treatment with efzimfotase alfa also resulted in dose-dependent reductions in the plasma PLP/PL ratio, which may better reflect ERT clinical efficacy in HPP patients than PLP concentrations alone.^20^ The observed reduction in plasma PPi, a potent inhibitor of bone mineralization, supports the potential therapeutic efficacy of efzimfotase alfa in improving bone mineralization, overall bone health, and physical function, as reported for asfotase alfa,^14^^,^^17^^,^^21^ with effects sustained for up to 7 yr.^22^ Efzimfotase alfa may also have potential as an ERT for infants and toddlers with life-threatening HPP, for whom asfotase alfa has been shown to improve pulmonary function and survival.^19^^,^^21^^,^^23^ Analyses of physical function (assessed by the 6-Min Walk Test among others), health-related quality of life, and other data from adults with HPP treated with asfotase alfa further confirmed the beneficial effects of the ERT in improving physical function and quality of life in a real-world setting.^24^^,^^25^

To avoid oversuppression of PPi, dosing regimens have been adjusted for the planned phase 3 clinical trials of efzimfotase alfa in children and adults with HPP (NCT06079281, NCT06079359, and NCT06079372) according to the following weight brackets, regardless of age: 10 to <30 kg: 20 mg s.c. every 2 wk (Q2W); 30 to <60 kg: 35 mg s.c. Q2W; and ≥ 60 kg: 50 mg s.c. Q2W. This dosing strategy was designed based on quantitative assessments via modeling and simulation to ensure that plasma efzimfotase alfa exposure and PPi reduction would be comparable with those observed in the asfotase alfa clinical trials in which there was no correlation between asfotase alfa exposure and incidence of ectopic calcification.^19^^,^^26^

The low treatment emergent anti-efzimfotase alfa ADA-positive rate and the absence of an apparent relationship between ADA positivity and efzimfotase alfa PK/PD further highlight the value of this ERT in the treatment of patients with HPP. However, these data should be interpreted with caution because of the small size and short duration of the study. Of note, such a conclusion is corroborated by the asfotase alfa first-in-human clinical trial, in which 2 of 6 (33%) participants tested positive for anti-asfotase alfa ADAs with 4 wk of treatment.^18^ The immunogenicity will be further evaluated in future studies on a larger cohort of patients treated with efzimfotase alfa for a longer duration.

The results of this study pave the way for informing the optimal efzimfotase alfa dosing regimens in future HPP clinical trials. However, the study has several limitations, including the small sample size, which was further reduced because of missed doses due to the COVID-19 pandemic, although the missing data did not appear to markedly affect PK or PD results. Additional limitations include the lack of a control group and relatively short study duration, as well as baseline plasma PPi concentrations that were within normal range, which may have resulted in significant portion of the PPi values being below the assay LLOQ after efzimfotase alfa treatment, particularly in the highest 90-mg dose cohort.

## Conclusion

This first-in-human phase 1 study has demonstrated that efzimfotase alfa, a second-generation ERT in clinical development for treating HPP, has acceptable safety, tolerability, and PK profiles, and achieved sustained reductions in biomarkers of disease in adults with HPP. These findings have informed the selection of appropriate dosing regimens in the planned phase 3 clinical trials of efzimfotase alfa for the evaluation and confirmation of the clinical safety and efficacy in both adult and pediatric patients with HPP.

## Supplementary Material

1850-Phase1_Manuscript-SUPPLEMENTARY_FIGURE_S1_zjae128

1850-Phase1_Manuscript-SUPPLEMENTARY_FIGURE_S2_zjae128

1850-Phase1_Manuscript-SUPPLEMENTARY_TABLE_S1_zjae128

1850-Phase1_Manuscript-SUPPLEMENTARY_TABLE_S2_zjae128

1850-Phase1_Manuscript-SUPPLEMENTARY_TABLE_S3_zjae128

1850-Phase1_Manuscript_Revised_7-16-24-SUPPLEMENTAL_MATERIAL_zjae128

## Data Availability

Alexion, AstraZeneca Rare Disease will consider requests for disclosure of clinical study participant-level data provided that participant privacy is assured through methods like data de-identification, pseudonymization, or anonymization (as required by applicable law), and if such disclosure was included in the relevant study informed consent form or similar documentation. Qualified academic investigators may request participant-level clinical data and supporting documents (statistical analysis plan and protocol) pertaining to Alexion-sponsored studies. Further details regarding data availability and instructions for requesting information are available in the Alexion Clinical Trials Disclosure and Transparency Policy at https://alexion.com/our-research/research-and-development. Link to Data Request Form: https://alexion.com/contact-alexion/medical-information.
