# Supplementary material for: Safety, pharmacokinetics, and pharmacodynamics of efzimfotase alfa, a second-generation enzyme replacement therapy: phase 1, dose-escalation study in adults with hypophosphatasia
Source: J Bone Miner Res. 2024 Aug 13;39(10):1412–23. doi: 10.1093/jbmr/zjae128 (PMC11425692; doi:10.1093/jbmr/zjae128)
Supplement: 1850-Phase1_Manuscript-SUPPLEMENTARY_FIGURE_S1_zjae128 [file 1850-phase1_manuscript-supplementary_figure_s1_zjae128.docx]

# Supplementary Materials

## Supplementary Figure 1. Participant Disposition


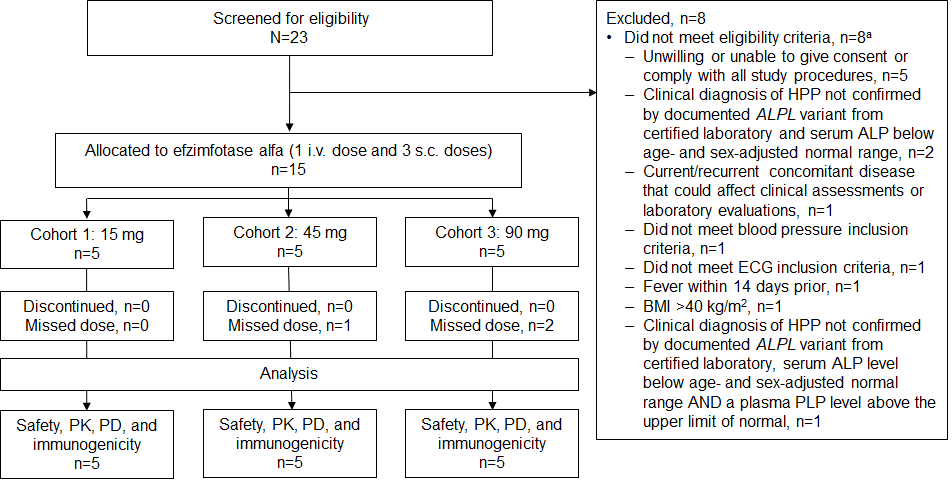


^a^Some participants met more than 1 exclusion criterion.

Note: Three participants missed doses because of asymptomatic COVID-19.

ALP, alkaline phosphatase; BMI, body mass index; ECG, electrocardiogram; HPP, hypophosphatasia; PD, pharmacodynamics; PK, pharmacokinetics; PLP, pyridoxal 5ʹ-phosphate.
