# Supplementary material for: Safety, pharmacokinetics, and pharmacodynamics of efzimfotase alfa, a second-generation enzyme replacement therapy: phase 1, dose-escalation study in adults with hypophosphatasia
Source: J Bone Miner Res. 2024 Aug 13;39(10):1412–23. doi: 10.1093/jbmr/zjae128 (PMC11425692; doi:10.1093/jbmr/zjae128)
Supplement: 1850-Phase1_Manuscript-SUPPLEMENTARY_FIGURE_S2_zjae128 [file 1850-phase1_manuscript-supplementary_figure_s2_zjae128.docx]

# Supplementary Materials

**Supplementary Figure 2.** Exploratory Outcomes

1. **Ionized calcium B. Phosphorus C. Magnesium**


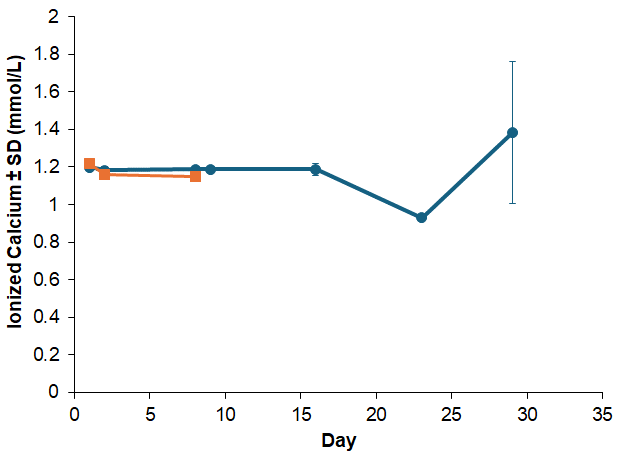

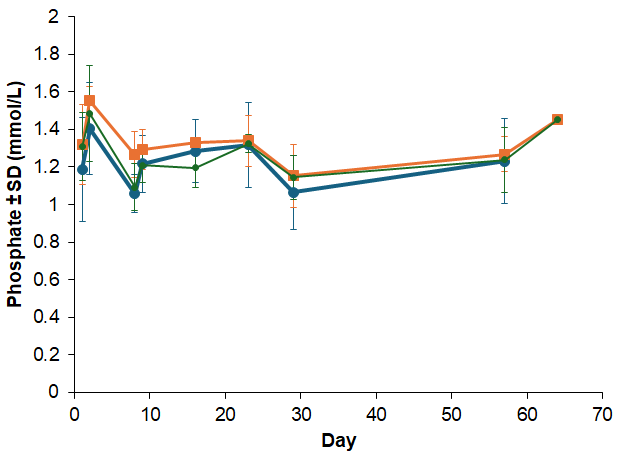

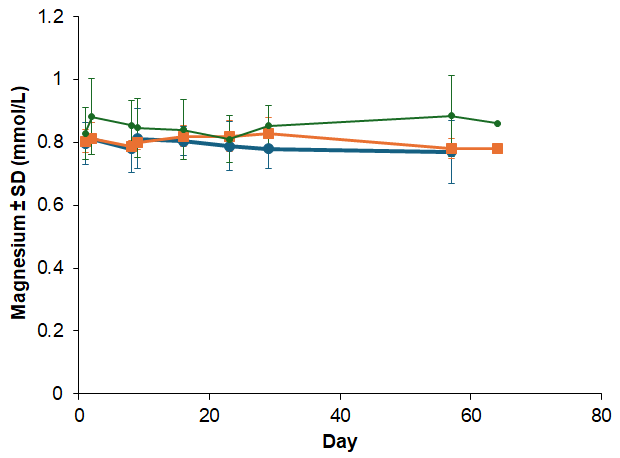


**D. Parathyroid hormone E. sCTX-1 F. P1NP**


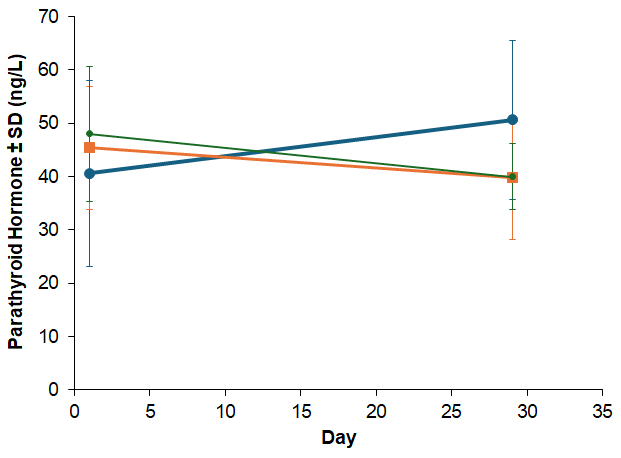

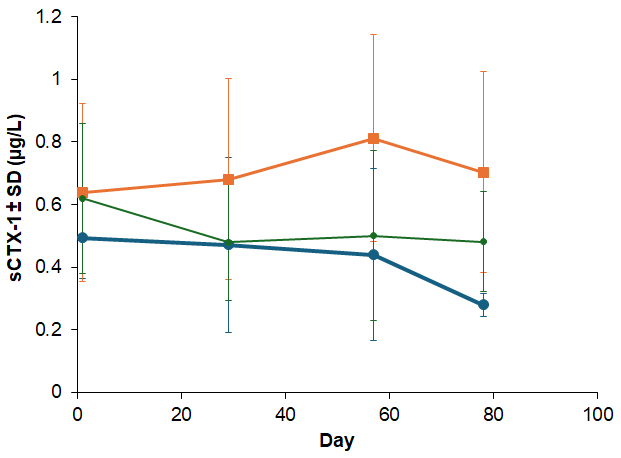

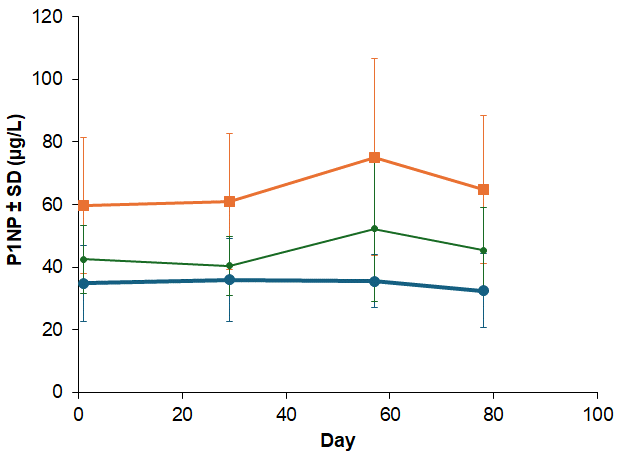


**G. Osteocalcin H. Pyridoxic Acid**


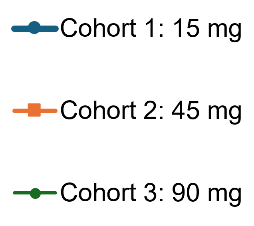

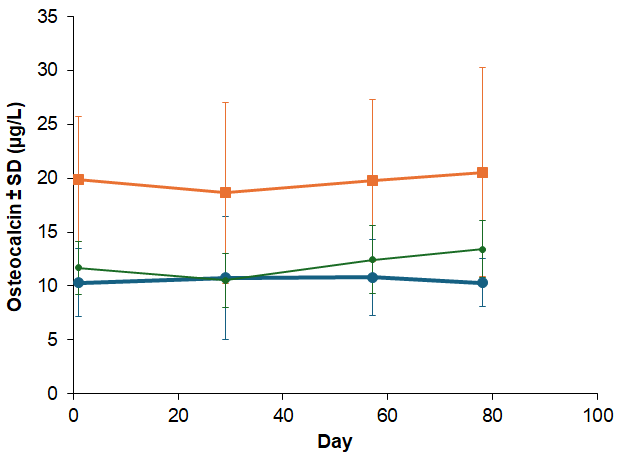

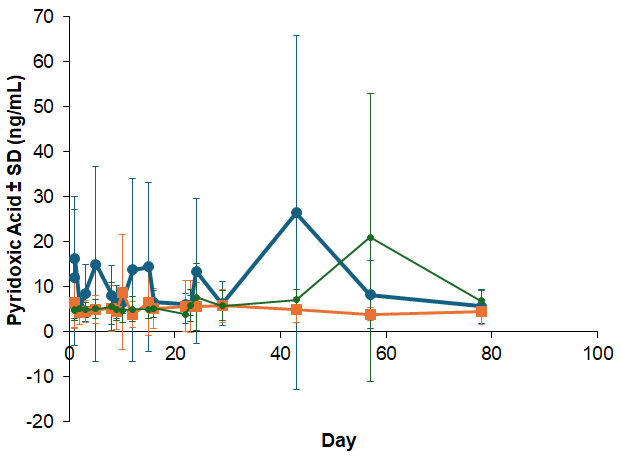


P1NP, N-terminal propeptide of type I procollagen; sCTX-1, serum C-terminal telopeptide of type 1 collagen; SD, standard deviation.
