# Supplementary material for: Safety, pharmacokinetics, and pharmacodynamics of efzimfotase alfa, a second-generation enzyme replacement therapy: phase 1, dose-escalation study in adults with hypophosphatasia
Source: J Bone Miner Res. 2024 Aug 13;39(10):1412–23. doi: 10.1093/jbmr/zjae128 (PMC11425692; doi:10.1093/jbmr/zjae128)
Supplement: 1850-Phase1_Manuscript-SUPPLEMENTARY_TABLE_S1_zjae128 [file 1850-phase1_manuscript-supplementary_table_s1_zjae128.docx]

# Supplementary Materials

**Supplementary Table 1. Statistical Assessment of Dose Proportionality of Efzimfotase Alfa Exposures Across the Dose Range of 15 to 90 mg**

| **Dose** | **PK parameter** | **n** | **Intercept** | **Estimated Slope (SE) [90% CI]** |
| --- | --- | --- | --- | --- |
| i.v. | C_max_ (μg/mL) | 15 | −1.07 | 0.995 (0.0975) [0.822, 1.17] |
|  | AUC_168_ (h•μg/mL) | 15 | 3.02 | 1.00 (0.100) [0.826, 1.18] |
|  | AUC_∞_ (h•μg/mL) | 13 | 3.29 | 0.984 (0.124) [0.761, 1.21] |
| s.c. 1 | C_max_ (μg/mL) | 12^a^ | −3.11 | 1.15 (0.170) [0.841, 1.46] |
|  | AUC_tau_ (h•μg/mL) | 12^a^ | 1.72 | 1.16 (0.169) [0.856, 1.47] |
| s.c. 2 | C_max_ (μg/mL) | 12^a^ | −3.68 | 1.22 (0.231) [0.802, 1.64] |
|  | AUC_tau_ (h•μg/mL) | 11^a^ | 1.33 | 1.20 (0.263) [0.718, 1.68] |
| s.c. 3 | C_max_ (μg/mL) | 11^a,b^ | −3.59 | 1.22 (0.333) [0.611, 1.83] |
|  | AUC_tau_ (h•μg/mL) | 11^a,b^ | 1.36 | 1.23 (0.326) [0.627, 1.82] |

^a^Analysis population was the subset of all participants who were not the first participant dosed in their respective cohort (i.e., subsequent participants).

^b^Data from the participant who missed s.c. dose 3 was excluded from analysis.

Abbreviations: AUC_∞_, area under the plasma concentration versus time curve from time 0 to time infinity; AUC_168_, area under the plasma concentration versus time curve from time 0 to 168 h; AUC_tau_, area under the plasma concentration versus time curve from time 0 to dosing interval; CI, confidence interval; C_max_, maximum observed plasma concentration; i.v. intravenous; s.c. subcutaneous.
