# Supplementary material for: Safety, pharmacokinetics, and pharmacodynamics of efzimfotase alfa, a second-generation enzyme replacement therapy: phase 1, dose-escalation study in adults with hypophosphatasia
Source: J Bone Miner Res. 2024 Aug 13;39(10):1412–23. doi: 10.1093/jbmr/zjae128 (PMC11425692; doi:10.1093/jbmr/zjae128)
Supplement: 1850-Phase1_Manuscript-SUPPLEMENTARY_TABLE_S2_zjae128 [file 1850-phase1_manuscript-supplementary_table_s2_zjae128.docx]

# Supplementary Materials

**Supplementary Table 2**. **Absolute Bioavailability of Efzimfotase Alfa Following s.c. Injection**

| **Efzimfotase alfa dose** | **Geometric LS Mean** | | **Ratio (s.c./i.v.) of Geometric LS Means (90% CI)** |
| --- | --- | --- | --- |
|  | **AUC_tau_ from s.c. Dose 3 (test)** | **AUC_∞_ from i.v. dose (reference)** |  |
| 15 mg | 112 (n=4) | 392 (n=3) | 0.286 (0.105, 0.782) |
| 45 mg | 367 (n=4) | 1000 (n=4) | 0.367 (0.180, 0.748) |
| 90 mg^a^ | 1070 (n=3) | 2920 (n=3) | 0.368 (0.0908, 1.49) |

^a^Data from participant who missed s.c. dose 3 were excluded from analysis.

Abbreviations: AUC_tau_, area under the plasma concentration versus time curve from time 0 to dosing interval; AUC_∞_, area under the plasma concentration versus time curve from time 0 to time infinity; CI, confidence interval; i.v. intravenous; LS, least squares; s.c. subcutaneous.

Data from the first participant in each cohort were excluded from the analysis.
