# Supplementary material for: Safety, pharmacokinetics, and pharmacodynamics of efzimfotase alfa, a second-generation enzyme replacement therapy: phase 1, dose-escalation study in adults with hypophosphatasia
Source: J Bone Miner Res. 2024 Aug 13;39(10):1412–23. doi: 10.1093/jbmr/zjae128 (PMC11425692; doi:10.1093/jbmr/zjae128)
Supplement: 1850-Phase1_Manuscript-SUPPLEMENTARY_TABLE_S3_zjae128 [file 1850-phase1_manuscript-supplementary_table_s3_zjae128.docx]

# Supplementary Materials

**Supplementary Table 3. Statistical Assessments of Pharmacokinetic Steady State of Efzimfotase Alfa**

| Cohort | Visit | n | Intercept | Slope (95% CI) | Steady state reached |
| --- | --- | --- | --- | --- | --- |
| Cohort 1 | Days 8, 15, and 22 | 4 | 0.839 | −0.0126 (−0.0149, 0.0166) | Yes |
| Cohort 2 | Days 8, 15, and 22 | 4 | 1.65 | 0.0239 (−0.0386, 0.0863) | Yes |
| Cohort 3 | Days 8, 15, and 22 | 4 | 4.50 | 0.0398 (−0.0376, 0.117) | Yes |

Abbreviation: CI, confidence interval.
