# Supplementary material for: Safety, pharmacokinetics, and pharmacodynamics of efzimfotase alfa, a second-generation enzyme replacement therapy: phase 1, dose-escalation study in adults with hypophosphatasia
Source: J Bone Miner Res. 2024 Aug 13;39(10):1412–23. doi: 10.1093/jbmr/zjae128 (PMC11425692; doi:10.1093/jbmr/zjae128)
Supplement: 1850-Phase1_Manuscript_Revised_7-16-24-SUPPLEMENTAL_MATERIAL_zjae128 [file 1850-phase1_manuscript_revised_7-16-24-supplemental_material_zjae128.docx]

# Supplementary Materials

## Supplementary Figure 1. Participant Disposition


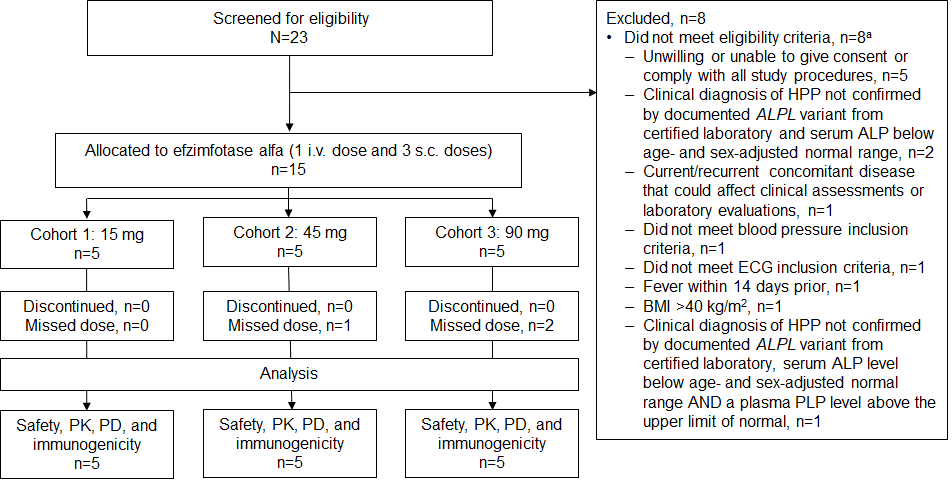


^a^Some participants met more than 1 exclusion criterion.

Note: Three participants were missed doses because of asymptomatic COVID-19.

ALP, alkaline phosphatase; BMI, body mass index; ECG, electrocardiogram; HPP, hypophosphatasia; PD, pharmacodynamics; PK, pharmacokinetics; PLP, pyridoxal 5ʹ-phosphate.

**Supplementary Figure 2.** Exploratory Outcomes

1. **Ionized calcium B. Phosphorus C. Magnesium**


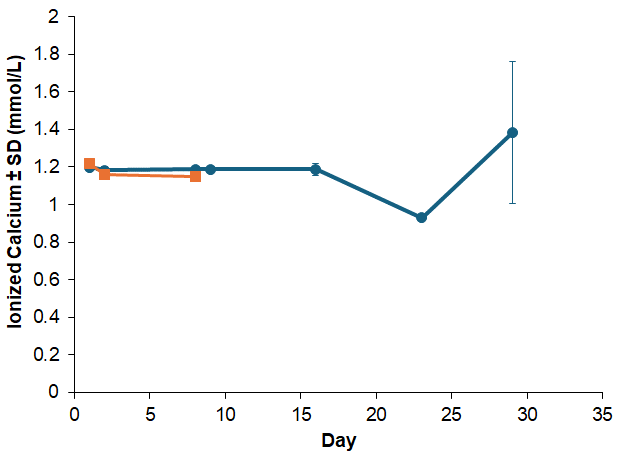

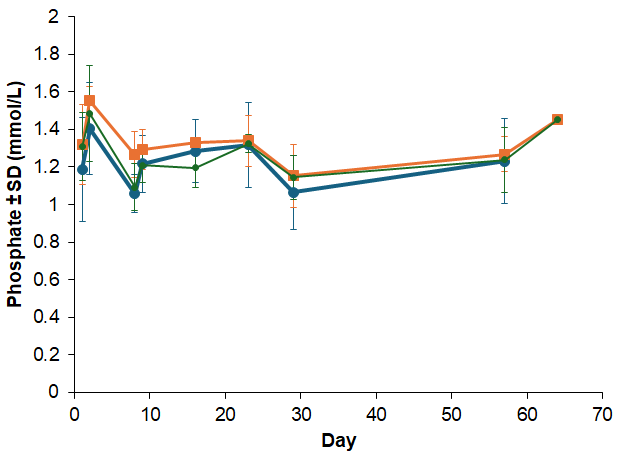

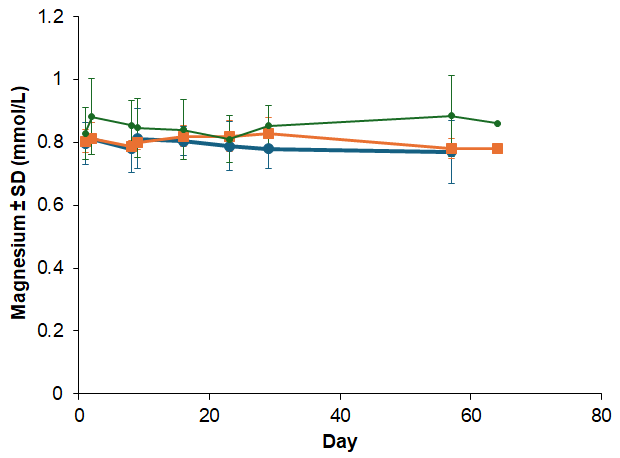


**D. Parathyroid hormone E. sCTX-1 F. P1NP**


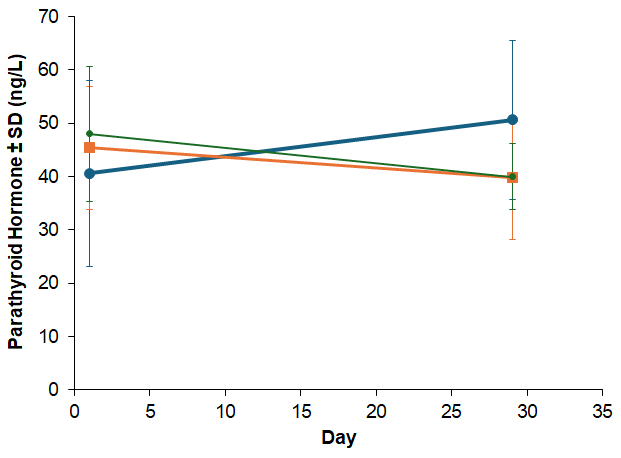

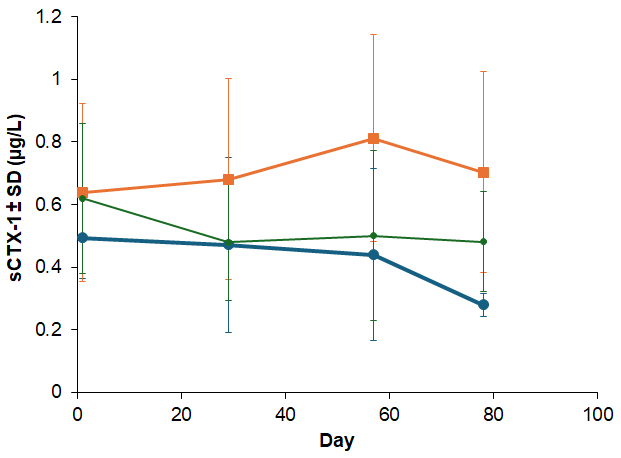

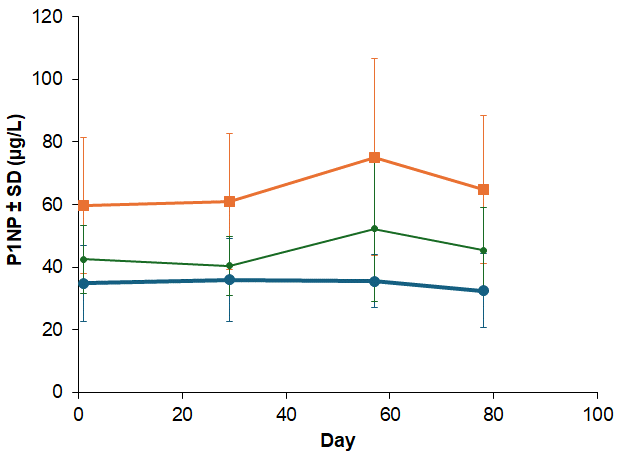


**G. Osteocalcin H. Pyridoxic Acid**


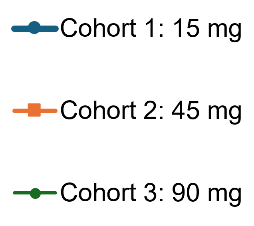

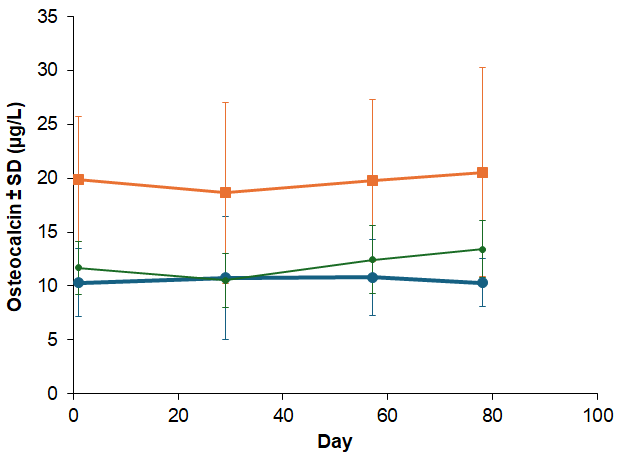

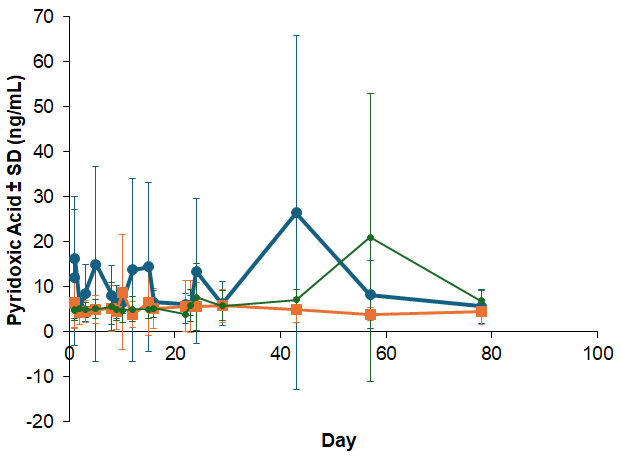


P1NP, N-terminal propeptide of type I procollagen; sCTX-1, serum C-terminal telopeptide of type 1 collagen; SD, standard deviation.

**Supplementary Table 1.** Statistical Assessment of Dose Proportionality of Efzimfotase Alfa Exposures Across the Dose Range of 15 to 90 mg

| **Dose** | **PK parameter** | **n** | **Intercept** | **Estimated Slope (SE) [90% CI]** |
| --- | --- | --- | --- | --- |
| i.v. | C_max_ (μg/mL) | 15 | −1.07 | 0.995 (0.0975) [0.822, 1.17] |
|  | AUC_168_ (h•μg/mL) | 15 | 3.02 | 1.00 (0.100) [0.826, 1.18] |
|  | AUC_∞_ (h•μg/mL) | 13 | 3.29 | 0.984 (0.124) [0.761, 1.21] |
| s.c. 1 | C_max_ (μg/mL) | 12^a^ | −3.11 | 1.15 (0.170) [0.841, 1.46] |
|  | AUC_tau_ (h•μg/mL) | 12^a^ | 1.72 | 1.16 (0.169) [0.856, 1.47] |
| s.c. 2 | C_max_ (μg/mL) | 12^a^ | −3.68 | 1.22 (0.231) [0.802, 1.64] |
|  | AUC_tau_ (h•μg/mL) | 11^a^ | 1.33 | 1.20 (0.263) [0.718, 1.68] |
| s.c. 3 | C_max_ (μg/mL) | 11^a,b^ | −3.59 | 1.22 (0.333) [0.611, 1.83] |
|  | AUC_tau_ (h•μg/mL) | 11^a,b^ | 1.36 | 1.23 (0.326) [0.627, 1.82] |

^a^Analysis population was the subset of all participants who were not the first participant dosed in their respective cohort (i.e., subsequent participants).

^b^Data from the participant who missed s.c. dose 3 was excluded from analysis.

AUC_∞_, area under the plasma concentration versus time curve from time 0 to time infinity; AUC_168_, area under the plasma concentration versus time curve from time 0 to 168 h; AUC_tau_, area under the plasma concentration versus time curve from time 0 to dosing interval; CI, confidence interval; C_max_, maximum observed plasma concentration; i.v. intravenous; s.c. subcutaneous.

**Supplementary Table 2**. **Absolute Bioavailability of Efzimfotase Alfa Following s.c. Injection**

| **Efzimfotase alfa dose** | **Geometric LS Mean** | | **Ratio (s.c./i.v.) of Geometric LS Means (90% CI)** |
| --- | --- | --- | --- |
|  | **AUC_tau_ from s.c. Dose 3 (test)** | **AUC_∞_ from i.v. dose (reference)** |  |
| 15 mg | 112 (n=4) | 392 (n=3) | 0.286 (0.105, 0.782) |
| 45 mg | 367 (n=4) | 1000 (n=4) | 0.367 (0.180, 0.748) |
| 90 mg^a^ | 1070 (n=3) | 2920 (n=3) | 0.368 (0.0908, 1.49) |

^a^Data from participant who missed s.c. dose 3 were excluded from analysis.

AUC_tau_, area under the plasma concentration versus time curve from time 0 to dosing interval; AUC_∞_, area under the plasma concentration versus time curve from time 0 to time infinity; CI, confidence interval; i.v. intravenous; LS, least squares; s.c. subcutaneous.

Data from the first participant in each cohort were excluded from the analysis.

**Supplementary Table 3. Statistical Assessments of Pharmacokinetic Steady State of Efzimfotase Alfa**

| Cohort | Visit | n | Intercept | Slope (95% CI) | Steady state reached |
| --- | --- | --- | --- | --- | --- |
| Cohort 1 | Days 8, 15, and 22 | 4 | 0.839 | −0.0126 (−0.0149, 0.0166) | Yes |
| Cohort 2 | Days 8, 15, and 22 | 4 | 1.65 | 0.0239 (−0.0386, 0.0863) | Yes |
| Cohort 3 | Days 8, 15, and 22 | 4 | 4.50 | 0.0398 (−0.0376, 0.117) | Yes |

CI, confidence interval.
